# IT adoption of clinical information systems in Austrian and German hospitals: results of a comparative survey with a focus on nursing

**DOI:** 10.1186/1472-6947-10-8

**Published:** 2010-02-02

**Authors:** Ursula Hübner, Elske Ammenwerth, Daniel Flemming, Christine Schaubmayr, Björn Sellemann

**Affiliations:** 1 Health Informatics Research Group, Faculty of Business Management and Social Sciences, University of Applied Sciences, Caprivistr. 30A, D-49076 Osnabrück, Germany; 2 Institute for Health Information Systems, UMIT - University for Health Sciences, Medical Informatics and Technology, Eduard Wallnöfer-Zentrum 1, A-6060 Hall/Tyrol, Austria; 3 Nursing Management, TILAK - Tiroler Landeskrankenanstalten, Anichstraße 35, A-6020 Innsbruck, Austria

## Abstract

**Background:**

IT adoption is a process that is influenced by different external and internal factors. This study aimed

1. to identify similarities and differences in the prevalence of medical and nursing IT systems in Austrian and German hospitals, and

2. to match these findings with characteristics of the two countries, in particular their healthcare system, and with features of the hospitals.

**Methods:**

In 2007, all acute care hospitals in both countries received questionnaires with identical questions. 12.4% in Germany and 34.6% in Austria responded.

**Results:**

The surveys revealed a consistent higher usage of nearly all clinical IT systems, especially nursing systems, but also PACS and electronic archiving systems, in Austrian than in German hospitals. These findings correspond with a significantly wider use of standardised nursing terminologies and a higher number of PC workstations on the wards (average 2.1 PCs in Germany, 3.2 PCs in Austria). Despite these differences, Austrian and German hospitals both reported a similar IT budget of 2.6% in Austria and 2.0% in Germany (median).

**Conclusions:**

Despite the many similarities of the Austrian and German healthcare system there are distinct differences which may have led to a wider use of IT systems in Austrian hospitals. In nursing, the specific legal requirement to document nursing diagnoses in Austria may have stimulated the use of standardised terminologies for nursing diagnoses and the implementation of electronic nursing documentation systems. Other factors which correspond with the wider use of clinical IT systems in Austria are: good infrastructure of medical-technical devices, rigorous organisational changes which had led to leaner processes and to a lower length of stay, and finally a more IT friendly climate. As country size is the most pronounced difference between Germany and Austria it could be that smaller countries, such as Austria, are more ready to translate innovation into practice.

## Background

Information technology supporting the work of physicians and nurses may help to improve patient safety, quality of care and organizational efficiency as has been summarized by different authors [e.g. [1,2]]. Despite the many positive effects IT systems can have the IT adoption rate in healthcare varies strongly between medical specialties [3], types of organisations [1,4,5] and countries. Surveys spanning many countries are traditionally conducted by international organisations such as the OECD, the WHO or the EU. They are meant to yield a broad picture of general topics identifying the leading countries and providing a profile per country. These studies are a rich source of data for gaining an up-to-date overview about different indicators. While the scope of some of these studies is rather general, e.g. the national healthcare systems and the health status [6], others are more focussed, e.g. covering eHealth [7] activities.

Besides multi-national, there are national [8] and bi-national studies [3]. In the United States the Healthcare Information and Management Systems Society (HIMSS) publishes regularly results of national IT surveys which give insight into the developments having taken place over the years [8]. These analyses describe IT trends at a meso-level, the level of different hospitals and their characteristics in one country.

In contrast, multi-national and bi-national studies on IT prevalence reveal factors influencing IT adoption that are associated with characteristics of the countries and their healthcare system (macro-level). Forces acting on this level have not been investigated extensively so far. Most of the studies on IT adoption cover healthcare providers in one country or in one region within this country (meso-level). Several factors are being discussed at this level to influence the decision whether a hospital adopts an IT system or not. Among these factors there are hospital size [9-12], system affiliation [10-12], teaching versus non teaching hospital [4,9-13], ownership [9-13], location (urban vs. rural area) [12] and IT budget and IT staff [4]. Only system affiliation was unanimously regarded [10-12] to exert an influence on IT adoption: It could be demonstrated that members of a health system or group of hospitals were more ready to implement IT systems than single hospitals. Also status as teaching hospital seems to positively influence [4,9-13] the willingness to use IT. Hospital size and ownership (for-profit, not-for-profit, government) are discussed controversially. Some authors found large [9] or not-for profit hospitals [9,13] to have more clinical IT systems than smaller or for-profit hospitals. Yet other studies did not find any (consistent) influence of hospital size or ownership on the prevalence of clinical IT systems [11,12]. Wang and colleagues [10] reported that more for-profit hospitals had managerial IT systems than not-for-profit hospitals. More IT staff and higher IT budgets were found to positively affect IT adoption [4] as well as location in an urban area [12].

IT adoption is also studied at the micro-level, i.e. within one hospital once a positive decision to purchase an IT system was made. Several theories have been proposed to understand factors leading to IT adoption. For example, the task-technology fit model [14] describes how the three factors tasks, technology and users interact and influence user evaluation of IT systems.

The following study aimed to compare the installation rates of clinical information systems, in particular of medical and nursing systems, in Austria and Germany. As geographical neighbours, who also share the same language, there are strong cultural bonds between the two countries, which among others are reflected by great similarities of the national healthcare systems and in the education and training of nurses. With regard to nursing education both countries are slowly changing their system for registered nurses from a diploma type of education to an academic programme with a Bachelor's degree.

The most salient difference is the country size (Table 1). According to the OECD study on healthcare [6], Austria and Germany share similar conditions with regard to healthcare expenditure and relative number of hospital beds, but they differ in length of stay. Indicators of the healthcare system that describe nursing, i.e. number of nurses and number of cases per nurse in a hospital, show only slight differences. Further statistics [15,16] reveal a difference in the relative number of large imaging devices in hospitals which is reflected e.g. by the number of MRI and CT units (Table 1).

**Table 1:** Selected indicators describing the healthcare systems in Austria and Germany

Indicator	Austria	Germany
Total population in Mio.+	**8.2**	**82.5**

Life expectancy at birth in years +	79.5	79.0

Total health expenditure as a share of Gross Domestic Product (GDP) in % +	10.2	10.7

Public and private health expenditure per capita in US $ +	3519.0	3287.0

Hospital beds per 1000 population +	6.1	6.4

Average length of stay (LOS) in days +	**5.9**	**8.6**

Magnetic Resonance Imaging (MRI) units per million population (hospitals only) ++	**10.1**	**8.0**

Computer Tomography (CT) units per million population (hospitals only) ++	**19.6**	**15.9**

Number of registered nurses per 100,000 population +++	6.1	7.6

Average number of cases per nurse in hospitals ++++	51.8	57.7

Differences printed in bold

**Sources: **+ [6], ++ [15,16], +++ [17], ++++ [18,19]

In 1997 Austria introduced a new system for financing hospitals, the leistungsbezogene Krankenhausfinanzierung - LKF, which replaced the diem based approach. The LKF system consists of "performance based groups" as well as of "diagnosis related groups" and is referred to as the "Austrian DRG system" [20]. Despite a general trend for a decreasing length of hospital stays already going on for several years in Austria, the LKF system accentuated the decrease. This influence was statistically significant and could be measured for various diagnostic groups [21]. In Germany a comparable case-based method started years later in 2004 when the German Diagnosis-Related-Group system (G-DRG system) was made compulsory for hospitals.

Studies on the use of electronic nursing documentation systems carried out in 2002/2003 in Germany [22] and Austria [23] showed an identical low degree of installations in acute care hospitals (about 7% of all hospitals). These similarities were surprising because there were striking differences in the legal requirements for nursing documentation between the two countries. The Austrian Healthcare and Nursing Act of 1997 [24] explicitly stipulates the documentation of nursing diagnoses, whereas comparable German laws only regulate the implementation of the nursing process in a very general manner. We were therefore interested whether there were any changes over the course of the time in the percentage of nursing documentation systems installed and if yes whether they would be parallel in both countries. In addition, we wanted to compare these observations with the adoption rate of electronic patient record (EPR) systems and other clinical systems within the hospital information system (HIS). Furthermore, we wanted to know if context variables of the hospitals, i.e. finances, IT services, IT infrastructure and user satisfaction, showed any differences in order to identify intervening factors. The objectives of this paper, therefore, are twofold:

1. to identify similarities and differences in the prevalence of medical and nursing IT systems in Austrian and German hospitals, and

2. to match these findings with characteristics of the two countries, in particular their healthcare system, and with features of the hospitals.

## Methods

We choose a cross-sectional study design to answer these questions. Against this background we conducted two surveys in Germany and Austria which used an identical questionnaire with eight clusters of questions covering the topics hospital demographics, IT infrastructure, EPR, nursing information systems in use and in planning, requirements of nursing information systems, financial situation, and access to the national eHealth infrastructure. Small changes to the questions had to be made due to country-specific terminologies and due to national peculiarities in eHealth. Comparable versions of this questionnaire had been already used in earlier surveys [22,25,26]. The current version of the questionnaire contained a total of 40 questions of which three questions were open-ended, the remaining 37 were closed. The terms "electronic patient record" and "nursing information system" were defined in the questionnaire because they do not have a generally accepted meaning. The questionnaire is available in an English version from http://www.it-report-healthcare.info and in the annex of this paper [additional file 1].

In both countries the questionnaire was sent to the nursing managers of all acute hospitals (2,172 hospitals in Germany, 130 hospitals in Austria). The survey period ranged from March to November 2007.

Statistical significance between the countries was calculated by χ^2^-tests for nominal data, by Mann-Whitney tests for ordinal and by t-tests for metric data. If metric data had outliers the Mann-Whitney test was used. When multiple tests were performed alpha was adjusted according to the Bonferroni method for avoiding the false appearance of significance. Alpha two sided was defined as 0.05 to consider the result of p ≤ α as significant which is denoted with *. Results where p ≤ 0.01 are marked with **, while those where 0.05 < p ≤ 0.1 with (*). Only when comparing the sample with the population we set alpha to 0.25 in order to minimize the beta error [27]. In the following, we will focus on the results of the main questions that means on IT infrastructure, EPR, nursing information systems in use and the financial situation.

## Results

### Sample

Hospitals of all sizes and from all federal states (Bundesländer) participated in Germany and Austria - with the exception of Mecklenburg-Pomerania, Germany where none of the hospitals took part. The return rate for Germany amounted to 12.4% (n = 270), for Austria to 34.6% (n = 45).

**Figure 1:**
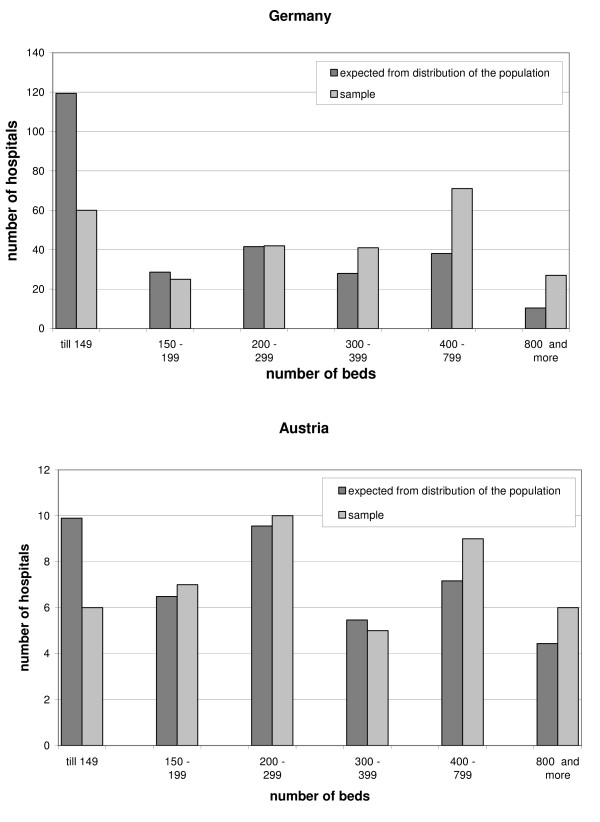
**Sample Population. **Sample and population distribution of the variable "hospital size" in Germany and Austria.

Whereas the sample distribution regarding hospital size did not differ significantly from the population (χ^2^-test) in Austria (p = 0.75), this difference was significant in the German sample (p = 0.00). Figure 1 shows the absolute number of hospitals in the two samples compared with those expected from the distribution of the population. In both countries geographic location in the samples varied significantly from the population (p_AUT _= p_GER _= 0.15 < 0.25). Between the two samples there were no significant differences in hospital size (p = 0.35), teaching status (p = 0.63) and system affiliation (p = 0.19).

### Clinical systems

**Figure 2:**
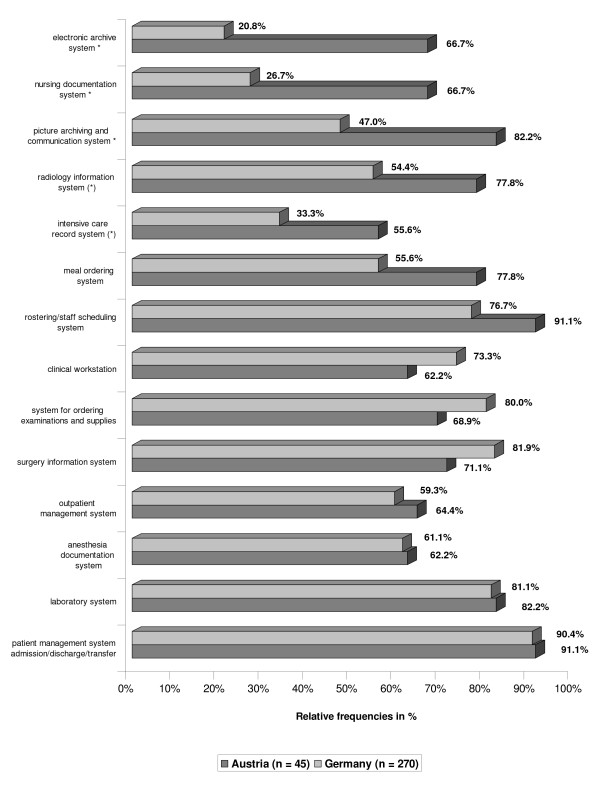
**IT systems. **IT systems in Austrian and German hospitals, in percentage of all respondents -- sorted by magnitude of difference between Austria and Germany.

Respondents were asked to indicate the IT systems available in their hospital from a list of 24 typical systems. Figure 2 shows the clinical systems most frequently mentioned by the respondents. As can be seen the majority of the systems was more widely used in Austria than in Germany, with significant difference for electronic nursing documentation systems, picture archiving and communication systems (PACS) and electronic archive systems (χ^2^-tests). In fact the difference in percentage points for nursing documentation systems was second highest - only surpassed by the electronic archive system. Systems that were already frequently used in Germany, such as patient management and laboratory systems, showed nearly identical values in both countries whereas for systems with a low prevalence in Germany the difference was very pronounced. There were no significant differences for administrative systems which all had high installation rates (70% and greater).

### Software related to nursing

**Figure 3:**
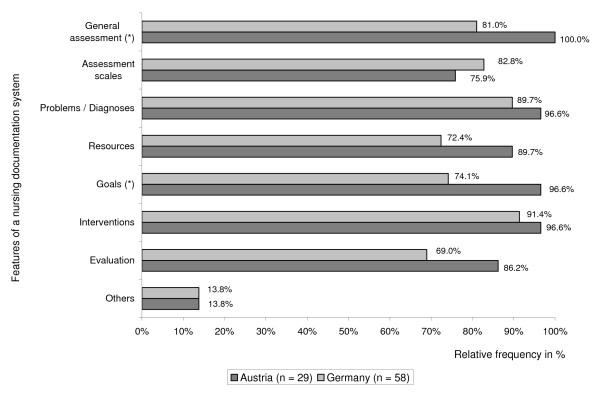
**Nursing Process. **Percentage of features of the nursing process supported by the nursing documentation systems in Austrian and German hospitals.

We also asked the respondents which steps of the nursing process were supported by the nursing documentation system. Typically the nursing process consists of the five phases: general assessment, nursing problem/diagnosis, goal, intervention planning and documentation and evaluation [28]. We furthermore asked the hospitals about the use of "assessment scales", documentation of "patient resources" and documentation of further nursing-related information ("others"). This adds up to eight steps to be supported by the nursing documentation system. Figure 3 shows the degree to which the nursing documentation systems in use covered the individual steps. At a descriptive level all steps but "assessment scales" and "others" were better represented in Austrian than in German systems, with "general assessment", "resources", "goals" and "evaluation" yielding the largest differences. The category "others" included among others entries for the documentation of wounds, falls and patients' need for care. After correcting alpha according to the Bonferroni method all differences missed significance.

**Figure 4:**
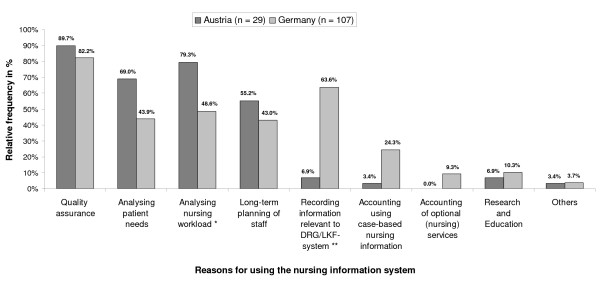
**Reasons. **Reasons why nursing information systems were used in Austria and Germany.

We were then interested to learn more about for what purpose the nursing system was used. Respondents were able to choose from a list of nine items (Figure 4). We did not restrict this question to nursing documentation systems but included all other types of nursing-related software which we then called nursing information system. This term was defined in the questionnaire to embrace all nursing related information used for patient care and administrative purposes. The findings (Figure 4) showed that in both countries its primary purpose was "quality assurance". Major differences between the countries concerned the reasons given besides "quality assurance". In Austria "analysing nursing workload" was the reason ranking second after "quality assurance" and it was significantly more often mentioned than in Germany. Also other staff related applications (measuring "patient needs" and "long-term planning of staff") were more frequently reported by Austrian than by German hospitals. However, these differences did not become significant.

In Germany the second ranking purpose was "recording information relevant to DRG/LKF-system". This difference between the two countries was significant (p ≤ 0.01).

In both countries most of the nursing documentation systems were integrated into the hospital information system (93.1% in AUT vs. 89.3% in GER) or into the electronic patient record (100% both) if the hospital had installed an EPR. There were slightly more hospitals in Austria reporting to have a fully operational EPR system than in Germany but the difference was not significant (Table 2). All participants were then asked to state the benefits they expected from introducing an EPR, giving them a list of eight possible benefits and a four-point Likert-scale from "very large" to "very small" benefit. The eight items were "influence on the quality of care", "improvement of the data quality", "availability of the data", "reduction of input errors", "reporting", "quality assurance", "research and education" and "increased staff satisfaction". For all these eight criteria, the percentage of answers in the category "very large" was higher for Austrian hospitals than for German hospitals. However, this difference was only significant for "data quality" (p = 0.004), where 55.6% of the Austrian hospitals valued data quality as "very large" as compared to 32.3% of the German hospitals.

**Table 2:** Status of the EPR in Austria and Germany

	fully operational EPR	installation begun	signed contract	developed plan to implement	no plan yet	do not know
**Austria (n = 42)**	11.9%	52.4%	0.0%	9.5%	23.8%	2.4%

**Germany (n = 268)**	7.0%	38.8%	2.7%	13.5%	34.9%	3.1%

Status categories adopted from HIMSS [8]

### Nursing terminologies

**Figure 5:**
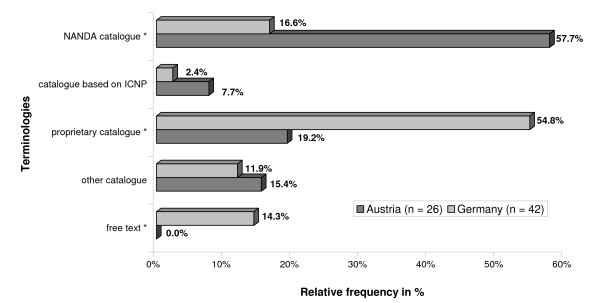
**Terminologies. **Nursing terminologies used for coding nursing diagnoses in % of respondents.

The respondents were asked which type of terminology they used for coding nursing diagnoses, resources, goals and interventions. As figure 5 shows the large majority of Austrian hospitals used the NANDA taxonomy for coding diagnoses whereas proprietary catalogues were the method of choice in Germany. In contrast to the diagnoses there were no significant differences between Austria and Germany with regard to resources, goals and interventions where entries were primarily coded with the help of proprietary catalogues in both countries.

### Context

The participants were also asked to give information on the context factors "central IT department", "nursing informatics (NI) specialist", "average number of PCs on the wards", average "IT budget" and average "user satisfaction" with the IT products installed (Table 3). Nearly all respondents both in Germany and Austria stated to have a central IT department (Table 3). Roughly half of them employed a nursing informatics (NI) specialist. On the average there were more PC workstations on the wards in Austrian than in German hospitals (p = 0.000). Austrian and German hospitals reported a nearly identical IT budget (no significant difference in Mann-Whitney test) of 2.6% of the yearly turnover in Austria and 2.0% in Germany (median). Overall user satisfaction with installed IT systems did not differ between the countries, although the average satisfaction on a 5-point Likert-scale (median) was higher in Austria - expressed by "satisfied" as compared to "neutral" in Germany.

**Table 3:** Context variables

	Central IT department in % (n)	Nursing informatics (NI) specialist in % (n)	Average number of PCs on ward ± SD (n)	Average IT budget median Q1/Q3 (n)	Average IT user satisfaction median Q1/Q3 (n)
**Austria**	93% (44)	47.7% (44)	3.2 * ± 1.3 (44)	2.6% 1.6%/4.2% (22)	"satisfied" "satisfied"/"neutral" (43)

**Germany**	90% (263)	45.9% (266)	2.1 * ± 1.1 (268)	2.0% 1.3%/5.0% (81)	"neutral" "satisfied"/"neutral" (253)

## Discussion

### Differences between the countries and their hospitals

The surveys revealed a consistent larger relative number of clinical IT systems in Austrian than in German hospitals. Among these results the large difference in nursing documentation systems is remarkable in particular as both countries started at a similarly low level of approximately 7% about 5 years ago [22,23]. The higher prevalence of nursing documentation systems in Austria goes well with the wide use of the NANDA taxonomy and other controlled vocabularies in Austria, because only electronic systems provide a suitable method for coding clinical data. The use of NANDA comes along with the obligations of the Austrian Healthcare and Nursing Act [24] which stipulates the documentation of nursing diagnoses. In both countries the nursing process is a procedure that is established by law. In contrast to Germany, the term "nursing diagnoses" is, however, explicitly used in the Austrian law only. This seems to have stimulated an entirely new clinical attitude towards the nursing process. Procedures how to make a nursing diagnosis based on clinical reasoning had to be established and nurses had to be trained accordingly. As we know from the literature diagnosing and translating the diagnoses into a terminology have a great impact on the way nurses work. Both require critical thinking and come along with improved documentation [29].

Stefan and Allmer's books [30,31] have served as a guideline for many hospitals in Austria not only to introduce the NANDA taxonomy but also to make nurses familiar with the procedure of diagnosing. In 2003 the results of theses efforts were hardly measurable [23]. Obviously it took several years before the process of establishing nursing diagnoses got going and substantial results could be observed. These developments demonstrate that mentioning the nursing process in laws - as it has been done in Germany - is not sufficient to stimulate change. It seems as if specific obligations can foster the implementation of new instruments more than general framework legislation as has been shown for quality management activities in three European countries [32].

On the other hand not only legal pressure led to more IT systems installed. The higher number of imaging devices in Austria [15,16] matches the finding of more PACS and RIS systems in use and may be regarded as an example for technical devices entailing the implementation of IT systems. With PACS in place, storing any other kind of patient documents electronically, including scanned paper documents, could become attractive for hospitals. This would explain the large difference in the number of installations of electronic archive systems.

IT adoption described at the macro-level thus seems to be a function of at least two variables: firstly legal demand - as demonstrated by the nursing documentation systems - and secondly a good infrastructure of medical-technical devices whose value can be enhanced by a new system - as shown by PACS and RIS. These two factors may influence the decision makers whether to acquire an IT system or not.

At the micro-level, IT adoption is the result of a subtle interaction between the fit of task, technology and user [2]. Whether the technology better fits the task in Austrian hospitals than in German hospitals is a matter to remain partly open. Only for nursing documentation systems this study allows some insight. It seems that the Austrian software or configuration of the software tends to be slightly more complete with regard to the phases of the nursing process. In Austria, partly other health IT products are marketed than in Germany, partly the same IT solutions [33]. However, this study does not provide any information on what products were actually installed in this sample. Previous surveys [22,25] had provided only unreliable information on this topic which hence had not been published. Therefore we did not ask for the specific products used.

The average user satisfaction with the systems installed was similarly high in our study, i.e. no significant difference could be found between the countries. This could be interpreted as an indicator for a sufficient user-technology-fit in both countries.

It therefore seems that characteristics at the micro-level are less likely to explain the differences in installation numbers.

Whereas the context variable average "average number of PCs on the ward" indicates more favourable conditions for clinical IT based applications, others show no difference between the two countries, e.g. nature of the IT support (existence of a central IT department and support provided by an NI specialist) and the IT budget. The "average number of PCs on the ward" can be interpreted as an indicator of the hospital's internal strategy to adopt IT as an instrument for supporting clinical processes and information accompanying these processes. In order to realise this strategy a sufficient number of PC workstations for data entry and retrieval is required. Both data entry and retrieval are tasks often performed by nurses. Our results show that nurses in Austria have a better infrastructure to accomplish them.

Among the variables characterizing hospitals, size is debated controversially in the literature [9-12] as a factor influencing IT adoption. In our study the significant differences between Austria and Germany, however, cannot be explained by differences in the size of the responding hospitals. Both samples showed a similar distribution of small, medium-sized and large hospitals without any significant variation. In a similar way the variables system affiliation [10-12] and teaching status [4,9-13], which are discussed to have an influence on the adoption of IT, could not have caused the differences between the two countries as the two samples did not differ with regard to these features. In conclusion, factors acting at the meso-level, i.e. properties of the hospitals do not provide sound explanations for the differences between the countries.

Obviously there are other forces promoting clinical IT. The considerably lower length of stay (LOS) in Austrian hospitals [6] hints at rigorous organisational changes which were already completed in Austria and which allow the hospital management to now turn to clinical matters once again after having completed the restructuring of the business processes. This particular focus on clinical IT systems is also reflected by the findings of a survey among Austrian hospitals [33] which showed that introducing clinical systems was given top priority in the next two years. Regarding length of stay and focus on clinical IT systems developments in Austria seem to resemble those in the United States where there was an average of 5.6 days as compared to 5.9 days in Austria 2005. In contrast, average length of stay in Germany was 8.6 days. With this value Germany was the country with the third highest LOS among OECD countries only surpassed by Japan and Korea. As the 15-year trend shows Germany had a long way to go from 14 days in 1990 to 8.6 days in 2005, whereas Austria ranged among the countries with lower LOS values already in 1990 (9.3 days) [6]. The case-based method of hospital financing which was introduced earlier in Austria than in German gave Austria another head start.

Another difference between the two countries concerns the organisation of hospitals. Austrian hospitals, which are operated at the level of federal states, negotiate as a large group with IT vendors and are therefore in a more favourable position than many German hospitals. Often decisions about the installation of a system apply to a group of hospitals which makes multiplication of IT experience easy and increases the number of installed systems.

Finally, a generally more positive climate for establishing the necessary infrastructure and using electronic systems as reflected by the e-Readiness index of 8.39 for Austria - as compared to 8.00 for Germany [34] - might tip the balance. Austria scored more highly in 4 out of 6 indicators, namely in "connectivity and technology infrastructure", "legal environment", "government policy and vision" and "consumer and business adoption" [34]. But not all studies reflect a greater adoption of IT in Austria. General practitioners (GP) in Germany use computers for more tasks than their Austrian colleagues [7].

### Limitations

A limitation of this study is the bias in both samples, i.e. the samples varied significantly from the population with regard to location in Austria and Germany and with regard to hospital size in Germany only. We rejected the null hypothesis (similarity of sample and population) on the basis of p ≤ 0.25 in order to keep the β-error, i.e. false acceptance of the null hypothesis, small. Other studies [35] were less conservative and rejected the null hypothesis only when p ≤ 0.05 which makes it easier to demonstrate similarity between sample and poplation. In conclusion to our approach we suggest the absolute values - not the differences between the countries - to be interpreted with caution.

Due to the nature of cross-sectional studies we cannot make any inferences on what is the cause and what is the effect.

### Methodological issues

Another source of potential errors could be the difference in response rates between Austria (34.6%) and Germany (12.4%). A possible reason might be the way the hospitals were recruited. In Austria, also personal contacts to nursing managers had been used to distribute and collect the questionnaires, while in Germany the contact was established by mail only. Response rates are subject to many factors not least to the number of external requests for participating in a study. In Germany, it seems as if the willingness of hospitals to respond is decreasing over the last years [22,25,26] which may be caused by the large number of questionnaires arriving at the hospitals per year (over-surveying). The crucial question, however, is whether this could have affected the differences in the data. An argument that definitely speaks against this assumption is the similarity in hospital size, system affiliation and teaching status in both samples.

High non-response rates may cause large non-response errors when the probability of not-responding is correlated with the phenomenon of interest [36], e.g. hospitals with a low number of clinical IT systems in our study could be less inclined to participate in the survey. This fact may cause limitations for interpreting the absolute values which then would tend to be too high. There are no indications to assume that this bias would act differently in Austria and in Germany. It would therefore distort very likely Austrian and German data similarly, i.e. into the same direction resulting in too high absolute values. Nevertheless, the comparison of the data should not be affected and should therefore provide valid results.

Another source of error is a low item non-response rate which relates to a single question or a group of questions. In our study this potential error may have affected the data about hospital finances. Only 31% of the German hospitals and 53% of the Austrian hospitals responded to the question about the IT budget. This low response rate is a well known fact [35] and it may be either due to an unwillingness to report the data or due to simply not knowing them. In either case the data must be treated with caution and must not at any rate be over interpreted. Not knowing the IT budget at the level of the hospital management could be an indicator for its non-existence. This again implies that no dedicated plans had been made for spending money on IT but rather had been subsumed under other cost centres such as technology or the department where the system was installed. So therefore the question might have been phrased in the wrong way.

### Comparison with other studies

To our knowledge, this study is the first of its kind comparing German and Austrian hospitals with regard to IT systems in hospitals. However, surveys studying the prevalence and use of clinical IT in each of the two countries separately had been conducted [33,37]. In Germany, the Wegweiser study [37] included - among others - information on the use of IT systems in German hospitals as of 2007. Those clinical IT systems that were phrased similarly in the Wegweiser study and in our study are listed in table 4 together with the relative frequencies in % of the respondents. The results are sorted by the magnitude of the differences in percentage points.

**Table 4:** Comparison of German studies on clinical IT systems

	Wegweiser study	our study
**nursing documentation system**	55.0%	26.7%

**surgery information system**	72.0%	81.9%

**laboratory system**	74.0%	81.1%

**PACS**	53.0%	47.0%

**electronic patient record system ^1^**	40.0%	45.8%

**staff scheduling system**	72.0%	76.7%

**RIS**	56.0%	54.4%

The Wegweiser study did not distinguish between different implementation phases of the electronic patient record. Therefore the percentage values of hospitals with a fully operational EPR and of those implementing an EPR in our study were added

The data show both consistencies as well as disparities in the values. Most striking is the large difference between the two studies with regard to nursing documentation systems, whereas the percentage values for RIS, staff scheduling, PACS and laboratory systems are more similar. In some cases our study provides the higher values (laboratory system and surgery information system), in others the Wegweiser study (nursing documentation system and PACS). The smaller differences might be due to chance, i.e. different types of hospitals in the samples, however, the value of 55% of the hospitals that reported to use IT based nursing document seems extremely high. In our study only 26.7% of the German hospitals said they were using a nursing documentation system. Our value had been reproduced by another study [38] which was conducted by the association of IT vendors in Germany (VHitG). It found that 25% of the hospitals were using a nursing documentation system.

In contrast to other terms the meaning of "nursing documentation" is less standardised in particular across different professions and may therefore lead to a different interpretation depending on whether the respondent is a nurse or a hospital manager. Within the nursing profession "nursing documentation systems" always refer to record keeping of the nursing process and related information [39]. Therefore the differences found between Austria and Germany in our study are very unlikely to be caused by a different interpretation of this term.

### Outlook

This study provides data for two countries with many commonalities but also with distinct differences. Our main findings in particular the influence of legal demand, of technical infrastructure of medical-technical devices, of the length of stay and of an IT friendly climate on clinical IT prevalence must be replicated both over time with the same countries as well as with other countries of similar characteristics. It also has to be investigated whether and how more IT, in particular for documenting the nursing process, actually has an impact on improved nursing outcomes.

## Conclusions

In conclusion, this study shows that despite many cultural similarities considerable differences in the use of clinical IT can exist. They might have been caused, among others, by legal constraints, by device infrastructure and by critical changes in the system (low LOS) as well as by a general IT friendly climate. Financial resources do not seem to be a crucial factor. The question remains whether smaller countries have indeed a greater power for translating innovation into practice. Characteristics of social networks in small and large countries should help to answer the question. If this is so larger countries are advised to promote the adoption of health information and communication technology at state (e.g. Bundesländer) rather than at national level.

## Competing interests

The authors declare that they have no competing interests.

## Authors' contributions

UH designed the study and the statistical analysis, supervised the construction of the questionnaire and investigated the literature. EA investigated the literature and background information and contributed to the data collection. CS contributed to the construction of the questionnaire, contributed to the data collection and investigated background information. DF constructed the questionnaire, contributed to the data collection, performed the statistical analysis and investigated background information. BS constructed the questionnaire, contributed to the data collection and performed the statistical analysis. UH drafted the manuscript including the final version of the manuscript which was revised and approved by all co-authors.

## Pre-publication history

The pre-publication history for this paper can be accessed here:

http://www.biomedcentral.com/1472-6947/10/8/prepub

## Supplementary Material

Additional file 1 **Questionnaire (English version)**. The file contains the English version of the questionnaire used in Germany and Austria. Click here for file
