# Supplementary material for: IT adoption of clinical information systems in Austrian and German hospitals: results of a comparative survey with a focus on nursing
Source: BMC Med Inform Decis Mak. 2010 Feb 2;10:8. doi: 10.1186/1472-6947-10-8 (PMC2830164; doi:10.1186/1472-6947-10-8)
Supplement: Additional file 1 — Questionnaire (English version). The file contains the English version of the questionnaire used in Germany and Austria. [file 1472-6947-10-8-S1.PDF]

University of Applied Sciences Osnabrück
Department of Business Management and Social Sciences
Health Informatics Research Group
Björn Sellemann
P.O. Box 19 40

D-49009 Osnabrück Germany

IT-Report

Healthcare

2007

Nursing in the Information Age

www.it-report-healthcare.info

Please read the questions carefully.

© U. Hübner / B. Sellemann / D. Flemming / E. Ammenwerth / C. Schaubmayr

University of Applied Sciences Osnabrück
Dept. Business Management and Social Sciences
Health informatics Research Group

Demographic data

1	**ZIP code, city, state**					
2	**Did you participate in the 2002 survey "Nursing in the Information Age"?**	**YES**		**NO**		
3	**What is your position in the hospital?**					
4	**Type of hospital** <categories are country-specific>	General hospital		Other hospital		Clinic
	(please specify)	hospital type A<br>hospital type B<br>hospital type C<br>other hospital				
5	**Ownership** <categories are country-specific>	not-for profit		for profit		other
6	**System affiliation**	stand-alone hospital		network hospital		
7	**Number of beds**	-49	50 -99	100 -149	150 -199	200 -299	300 -399
		400 -499	500 -599	600 -799	800 -999	1000 and larger	

8	**IT-systems within the hospital information system (HIS)**	accounting	controlling	human resources	materials management	asset manage-ment	medical controlling
	**multiple answers possible**	patient-management (adm/dis/trans)	clinical work-station	ordering supplies & examinations	intensive care record	nursing docu-mentation	minimum data set
	**multiple answers possible**	surgery infor-mation	anesth. docu-mentation	laboratory	radiology information	PACS[2]	electronic archive
	**multiple answers possible**	outpat. manage-ment	staff sche-duling	time recording	quality management	meal ordering	device management
	**multiple answers possible**	others				others	
	**multiple answers possible**						

[1] Diagnosis Related Group
[2] Picture Archiving and Communication System

© U. Hübner / B. Sellemann / D. Flemming / E. Ammenwerth / C. Schaubmayr

University of Applied Sciences Osnabrück
Dept. Business Management and Social Sciences
Health informatics Research Group

9 **The HIS is an all-in-one solution.** — **NO** ☐ / **YES** ☐ ______ vendor

(continue with **9a-b**)

9a **The clinical system is an all-in-one solution.** — **NO** ☐ / **YES** ☐ ______ vendor

9b **The administrative system is an all-in-one solution.** — **NO** ☐ / **YES** ☐ ______ vendor

______

10 **How satisfied are you with the IT-products and services?**	very satisfied	satisfied	neutral	dissatisfied	very dissatisfied
	☐	☐	☐	☐	☐

IT-infrastructure

11 **Is there a central IT-department in your hospital?**	**NO**	**YES**
	☐	☐

12 **Is there a nursing informatics specialist in your hospital?**	**NO**	**YES**
	☐	☐

13 **What is the average number of PCs on the wards, e.g. desktops, notebook?**	**1**	**2**	**3**	**4**	**5**	**more**
	☐	☐	☐	☐	☐	______ number

14 **What is the most significant barrier to successfully implementing IT for NURSING in your institution today?**

Please select only ONE line.

Lack of strategic IT plan	☐
Lack of IT staff	☐
Lack of a nursing informatics specialist	☐
Difficulty in proving IT quantifiable benefits / return on investment	☐
Lack of common data standards	☐
Lack of support from hospital executives	☐
Difficullty in achieving end-user acceptance or use	☐
Lack of adequate financial support for IT	☐
Vendor's inability to effectively deliver product or service to our satisfaction	☐
Others	☐
Don't know	☐

Select only one line please.

© U. Hübner / B. Sellemann / D. Flemming / E. Ammenwerth / C. Schaubmayr

University of Applied Sciences Osnabrück
Dept. Business Management and Social Sciences
Health informatics Research Group

Electronic Patient Record*

15

**Please describe the status of your institution's current use of an electronic patient record (EPR) system.**	We have a fully operational EPR system in place.	We have begun to install EPR software and hardware.	We already signed a contract.	We have developed a plan to implement an EPR system	We have not yet begun to plan for the use of an EPR system.	Do not know
	☐	☐	☐	☐	☐	☐

16

**What is the potential benefit of implementing an EPR system* and how large is it?**	very large	large	small	very small	do not know
influence on the quality of care (e.g. by increasing the information exchange)	☐	☐	☐	☐	☐
improvement of the data quality (e.g. by avoidance of double captures)	☐	☐	☐	☐	☐
availability of the data (e.g. simultaneous access to the EPR by different healthcare professionals)	☐	☐	☐	☐	☐
reduction of input errors (e.g. by given data types (e.g. date) or integrity checks)	☐	☐	☐	☐	☐
reporting (e.g. medical and nursing summaries)	☐	☐	☐	☐	☐
quality assurance (e.g. monitoring and analysing of quality indicators)	☐	☐	☐	☐	☐
education / research (e.g. retrieval of patient records for research purposes)	☐	☐	☐	☐	☐
increased staff satisfaction (e.g. reduction of extra work such as retrieval of patient records)	☐	☐	☐	☐	☐

Please answer every question.

Please answer every question.

Nursing information system**

**** A nursing information systems includes the legally required documentation of the nursing process. It furthermore allows the analysis of quality data and economic nursing data for management purposes. It may consist of one or more subsystems of a hospital information system. *(after Schrader, U. and Ammenwerth, E., 2004)***

17 **Do you use IT-modules of a nursing information system?**

- ☐ **YES** — **Please continue with question <u>18.</u>**
- ☐ **NO** — **Please continue with question <u>29</u>.**

18

**Is the nursing documentation system integrated in the HIS?**	**YES**	**NO**
IF YES please continue with 18a.	☐	☐

18a

**Is the nursing documentation system integrated in the EPR?**	**YES**	**NO**
	☐	☐

19

**Is the staff scheduling system integrated in the HIS?**	**YES**	**NO**
	☐	☐

* **EPR Definition**: An EPR is an electronically originated and maintained clinical health information system, delivered from multiple sources, about an individuals lifetime health status and health care. An EPR is supported by clinical decision systems and replaces the paper medical record as the primary source of patient information.

© U. Hübner / B. Sellemann / D. Flemming / E. Ammenwerth / C. Schaubmayr

20	The software supports the following phases of the nursing process, i.e. documentation of …	general assessment	assessment scales	diagnoses / problems	resources
	multiple answers possible	☐	☐	☐	☐
		goals	interventions	evaluation	others
	multiple answers possible	☐	☐	☐	______ please specify

21	The following scales are integrated …	Braden-scale	Norton-scale	Barthel-index	FIM[4]	others
	multiple answers possible	☐	☐	☐	☐	______ please specify

22	Problems and diagnoses are described/coded by …	free text	propriet. cata-logue	catalogue based on the ICNP[5]	NANDA[6] catalogue	others
	Please choose one item only.	☐	☐	☐	☐	______ please specify

23	Resources are described/coded by …	free text	propriet. cata-logue	catalogue based on the ICNP[5]		others
	Please choose one item only.	☐	☐	☐		______ please specify

24	Interventions are described/coded by …	free text	propriet. cata-logue	catalogue based on the ICNP[5]	LEP[7] Nursing **2**	LEP[7] Nursing **3**
	Please choose one item only.	☐	☐	☐	☐	☐
		others	______ please specify			

25	Goals and outcomes are described/coded by …	free text	propriet. cata-logue	catalogue based on the ICNP[5]		others
	Please choose one item only.	☐	☐	☐		______ please specify

26	Data are entered using …	PCs on the ward	computer on wheels	PDAs[8] e.g. Palm	Barcode device at point of care	special device at point of care
	multiple answers possible	☐	☐	☐	☐	☐
		others	______ please specify			

[4] Functional Independence Measure (FIM)
[5] International Classification of Nursing Practice (ICNP)
[6] North American Nursing Diagnosis Association (NANDA)
[7] Leistungserfassung in der Pflege (LEP)
[8] Personal Digital Assistent (PDA)

© U. Hübner / B. Sellemann / D. Flemming / E. Ammenwerth / C. Schaubmayr

University of Applied Sciences Osnabrück
Dept. Business Management and Social Sciences
Health informatics Research Group

27 **The nursing information system is used for …**

multiple answers possible

quality assurance	analysing patient needs	analysing nursing workload	Long-term planning of staff	recording information relev. to DRG/LKF syst.
☐	☐	☐	☐	☐

multiple answers possible

account. using case-based nursing inform.	accounting of optional nursing services	research / education	others
☐	☐	☐	______ please specify

28 **Are you using LEP[7] (Nursing 2 oder 3)?**

(IF YES please continue with **28a-e**)

**YES**	**YES, but we are still implementing LEP.**	**NO**
☐	☐	☐

If YES, please answer every question.

LEP[7] is used for …

		**YES**	**NO**
28a	analysing the nursing workload	☐	☐
28b	short-term planning of staff	☐	☐
28c	long-term planning of staff	☐	☐
28d	controlling in nursing	☐	☐
28e	analysis of the C-value	☐	☐

Please answer every question.

Plans about implementing software used by nurses

(Please answer the question regardless of whether you are already using a nursing information system or not.)

29 **We plan to implement the following IT-systems**

	this year	in two years	yes, but not specified when	no	do not know
staff scheduling	☐	☐	☐	☐	☐
quality management	☐	☐	☐	☐	☐
patient management	☐	☐	☐	☐	☐
nursing documentation	☐	☐	☐	☐	☐
nursing workload analysis	☐	☐	☐	☐	☐
surgery information	☐	☐	☐	☐	☐
anesthesia documentation	☐	☐	☐	☐	☐
intensive care record	☐	☐	☐	☐	☐
ordering supplies and examinat.	☐	☐	☐	☐	☐
time recording	☐	☐	☐	☐	☐
meal ordering	☐	☐	☐	☐	☐
others	☐	☐	☐	☐	☐

© U. Hübner / B. Sellemann / D. Flemming / E. Ammenwerth / C. Schaubmayr

University of Applied Sciences Osnabrück
Dept. Business Management and Social Sciences
Health informatics Research Group

*Requirements of a good nursing information system***

(Please answer the question regardless of whether you are already using a nursing information system or not.)

Please fill in every line.

30 **The software should support the following phases of the nursing process, i.e. documentation of …**	very important	important	less important	not important	do not know
general assessment	☐	☐	☐	☐	☐
assessment scales	☐	☐	☐	☐	☐
nursing diagnoses / problems	☐	☐	☐	☐	☐
resources	☐	☐	☐	☐	☐
goals	☐	☐	☐	☐	☐
interventions	☐	☐	☐	☐	☐
evaluation	☐	☐	☐	☐	☐
others:______________________	☐	☐	☐	☐	☐

Please fill in every line.

31 **Problems and diagnoses should be described/coded by …**	free text	propriet. cata-logue	catalogue based on the ICNP[5]	NANDA[6] catalogue	others
**Please choose one item only.**	☐	☐	☐	☐	______ please specify

32 **Resources should be described/coded by …**	free text	propriet. cata-logue	catalogue based on the ICNP[5]		others
**Please choose one item only.**	☐	☐	☐	☐	______ please specify

33 **Interventions should be described/coded by …**	free text	propriet. cata-logue	catalogue based on the ICNP[5]	LEP[7] Nursing **2**	LEP[7] Nursing **3**
**Please choose one item only.**	☐	☐	☐	☐	☐

others: ______ please specify

34 **Goals and outcomes should be described/coded by …**	free text	propriet. cata-logue	catalogue based on the ICNP[5]		others
**Please choose one item only.**	☐	☐	☐	☐	______ please specify

35 **The software should include clinical pathways for …**	**YES**	**NO**
(If YES, please continue with **35 a-d**)	☐	☐

If YES, please answer every question.

	**YES**	**NO**
35a **processs descriptions and to-do-lists**	☐	☐
35b **documentation**	☐	☐
35c **steering the processes**	☐	☐

35d **other purposes:** ______________________

34 **Data should be entered using …**	PCs on the ward	computer on wheels	PDAs[8] e.g. Palm	Barcode device at point of care	special device at point of care
**multiple answers possible**	☐	☐	☐	☐	☐

others: ______ please specify

© U. Hübner / B. Sellemann / D. Flemming / E. Ammenwerth / C. Schaubmayr

University of Applied Sciences Osnabrück
Dept. Business Management and Social Sciences
Health informatics Research Group

Please fill in every line.

35

**The nursing information system should be used for …**	very important	important	less important	not important	do not know
quality assurance	☐	☐	☐	☐	☐
analysing patient needs	☐	☐	☐	☐	☐
analysing the nursing workload	☐	☐	☐	☐	☐
long-term staff scheduling	☐	☐	☐	☐	☐
short-term staff scheduling	☐	☐	☐	☐	☐
recording information relevant to DRG/LKF system	☐	☐	☐	☐	☐
accounting using case-based nursing information	☐	☐	☐	☐	☐
accounting of optional nursing services	☐	☐	☐	☐	☐
research	☐	☐	☐	☐	☐
other:______________________	☐	☐	☐	☐	☐

Please fill in every line.

Access to eHealth applications

36

**How important is the access to patient data in eHealth applications for nurses …**	very important	important	less important	not important	do not know
	☐	☐	☐	☐	☐

37

**<This question was country specific.>**	**YES**	**NO**
Option A	☐	☐
Option B	☐	☐

38 **<This question was country-specific.>**

☐ ☐ ☐

Finances

39

**How does the DRG/LKF-system influence the economic situation of your hospital?**	positively	negatively	not at all
	☐	☐	☐

40 **What is your IT-Budget (as percent of your yearly turnover)?** ________ %

Thank you for your time.

© U. Hübner / B. Sellemann / D. Flemming / E. Ammenwerth / C. Schaubmayr
